# Effect of Light and Dark on the Phenolic Compound Accumulation in Tartary Buckwheat Hairy Roots Overexpressing *ZmLC*

**DOI:** 10.3390/ijms22094702

**Published:** 2021-04-29

**Authors:** Chang Ha Park, Ye Eun Park, Hyeon Ji Yeo, Nam Il Park, Sang Un Park

**Affiliations:** 1 Department of Crop Science, Chungnam National University, 99 Daehak-ro, Yuseong-gu, Daejeon 34134, Korea; parkch804@gmail.com (C.H.P.); yeney1996@cnu.ac.kr (Y.E.P.); guswl7627@gmail.com (H.J.Y.); 2 Department of Plant Science, Gangneung-Wonju National University, 7 Jukheon-Gil, Gangneung 25457, Korea; nipark@gwnu.ac.kr; 3 Department of Smart Agriculture Systems, Chungnam National University, 99 Daehak-ro, Yuseong-gu, Daejeon 34134, Korea

**Keywords:** *Fagopyrum tataricum*, *ZmLC*, phenolic compounds

## Abstract

*Fagopyrum tataricum* ‘Hokkai T10′ is a buckwheat cultivar capable of producing large amounts of phenolic compounds, including flavonoids (anthocyanins), phenolic acids, and catechin, which have antioxidant, anticancer, and anti-inflammatory properties. In the present study, we revealed that the maize transcription factor Lc increased the accumulation of phenolic compounds, including sinapic acid, 4-hydroxybenzonate, *t*-cinnamic acid, and rutin, in Hokkai T10 hairy roots cultured under long-photoperiod (16 h light and 8 h dark) conditions. The transcription factor upregulated phenylpropanoid and flavonoid biosynthesis pathway genes, yielding total phenolic contents reaching 27.0 ± 3.30 mg g^−1^ dry weight, 163% greater than the total flavonoid content produced by a *GUS*-overexpressing line (control). In contrast, when cultured under continuous darkness, the phenolic accumulation was not significantly different between the *ZmLC*-overexpressing hairy roots and the control. These findings suggest that the transcription factor (ZmLC) activity may be light-responsive in the *ZmLC*-overexpressing hairy roots of *F. tataricum*, triggering activation of the phenylpropanoid and flavonoid biosynthesis pathways. Further studies are required on the optimization of light intensity in *ZmLC*-overexpressing hairy roots of *F. tataricum* to enhance the production of phenolic compounds.

## 1. Introduction

*Fagopyrum tataricum* (Tartary buckwheat) is a common specialty crop with a bitter flavor that has been actively consumed for its various bioactive compounds, such as vitamins, flavonoids, amino acids, and proteins [1]. Additionally, the intake of Tartary buckwheat is considered beneficial as previous studies have identified a variety of strong biological and pharmacological properties, including antioxidant and anti-inflammatory effects, diabetic control, and carcinogenesis and tumor inhibition [2,3,4].

*Agrobacterium rhizogenes* is considered a natural plant genetic engineer that can induce transgenic hairy roots, in the form of adventitious roots, by transferring the T-DNA region on the root-inducing (Ri) plasmid to the host plant genome [5]. The hairy roots from mother plants have been shown to sustain vigorous growth without exogenous phytohormone application compared with their non-transgenic counterparts [6]. They also have the ability to produce the secondary metabolites present in the mother plant [7], often in greater quantity than in the parent [7]. Furthermore, *A. rhizogenes*-mediated hairy roots have been utilized for transgenic plant development, the increase in phytochemical production, and the investigation of metabolic processes [8].

Leaf Color (LC) is a regulatory protein in *Zea mays* involved in anthocyanin biosynthesis [9]. Ectopic expression of this transcription factor in various plant species results in anthocyanin accumulation and the related purple color in transgenic plants, as well as an increase in the contents of other phenolic compounds, including phenolic acids and flavonoids. For example, exogenous expression of *ZmLC* enhanced phenolic compounds by regulating flavonoid biosynthesis in the flowers of tobacco [10] and the leaves of apples [11]. Additionally, Park et al. reported that *ZmLC* overexpression in *Scutellaria baicalensis* hairy roots increased in baicalin, baicalein, and wogonin (a flavone group), with activation of the phenylpropanoid biosynthesis pathway [12]. However, in several species, *ZmLC* did not have a strong effect [13,14], whereas co-expression with *Pl* (*purple leaf*) ultimately enhanced anthocyanin accumulation; *ZmLC* expression returned no visible effect in creeping bentgrass [15]. Interestingly, anthocyanin accumulation was reported to be light-dependent in *ZmLC*-transgenic maize seeds [16], as well as in petunia [17], alfalfa [18], and cotton leaves [19].

**Figure 1:**
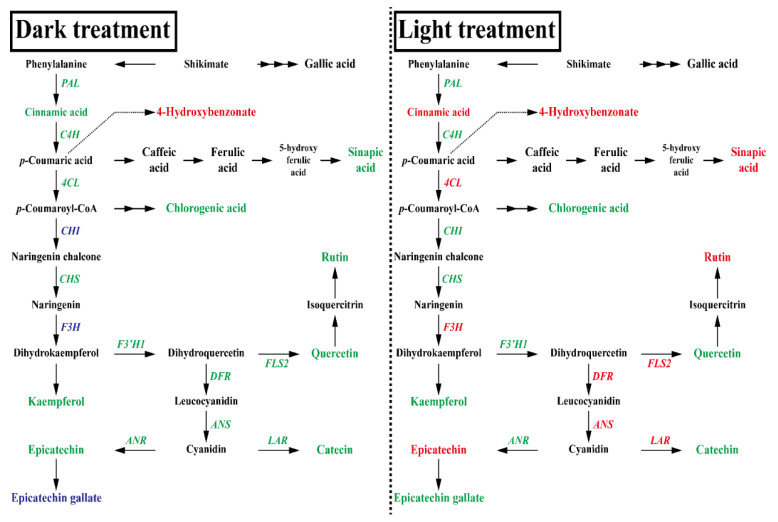
Proposed biosynthetic pathways for phenylpropanoid and flavonoids in Tartary Buckwheat hairy roots overexpressing *ZmLC* after dark and light treatment, respectively. Italicized red and blue text indicate significantly upregulated and downregulated genes, respectively (*p* < 0.05), after dark and light treatment, respectively, whereas the italicized green text indicates genes that failed to show any significant differences. Red and blue text indicate a significant decrease and increase in phenolic compounds, respectively (*p* < 0.05), after dark and light treatment, respectively, whereas the green text indicates phenolic compounds that failed to show any significant differences.

Previous studies have reported that Tartary buckwheat hairy roots are viable for phenolic compound production because of the high concentration in these phenolics [20,21]. However, there is little research on the effect of *ZmLC* overexpression on flavonoid biosynthesis in the hairy roots of this species. In this study, we induced *ZmLC*-transgenic hairy roots from Tartary buckwheat to enhance the accumulation of phenolics and investigated the effect of hairy root culture under both long photoperiod (16 h light and 8 h dark) conditions and continuous darkness on phenolic compound accumulation (Figure 1).

## 2. Results

### 2.1. Hairy Root Induction

**Figure 2:**
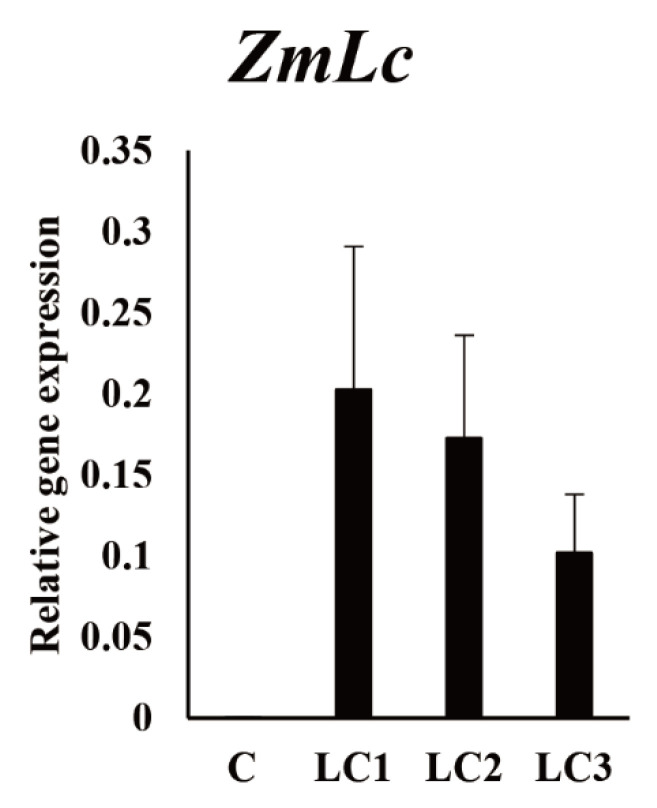
qRT-PCR analysis for *ZmLC* of *GUS*- and *ZmLC*-overexpressing hairy root lines. C: *GUS*-overexpressing hairy root line; LCn: *ZmLC*-overexpressing hairy root lines.

qRT-PCR was performed to measure *ZmLC* expression in the *GUS*- and three -transgenic hairy root lines. *ZmLC*-transgenic hairy root lines showed higher *ZmLC* expression than in the *GUS* line, verifying the production of one and three independent transgenic Tartary buckwheat hairy root lines overexpressing *GUS* and *ZmLC*, respectively (Figure 2).

### 2.2. Effect of Dark Treatment on the Production of Phenolic Compounds in ZmLC- and GUS-Transgenic Hairy Root Lines of Tartary Buckwheat

**Figure 3:**
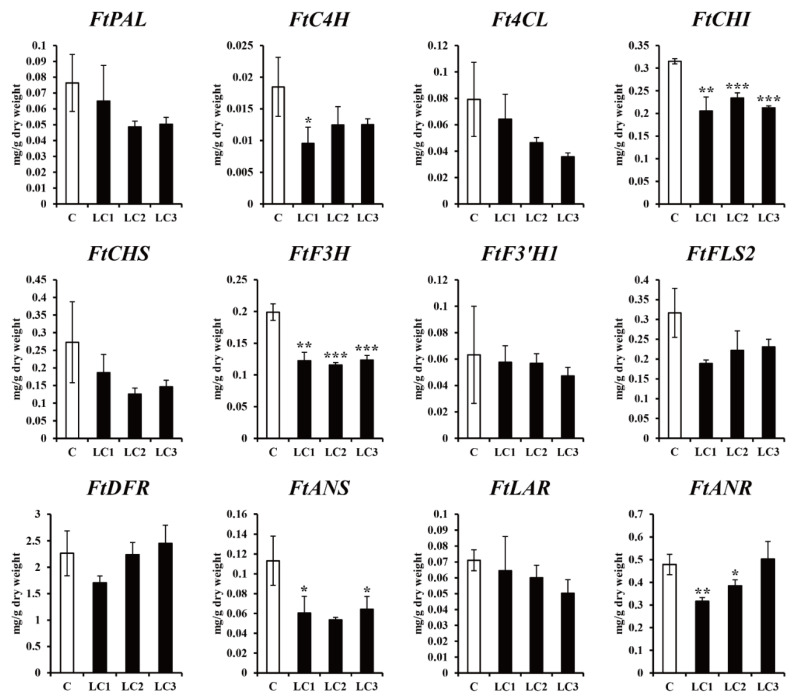
Effect of dark treatment on the expression profiles of hairy root cultures in *Fagopyrum tataricum*. C: *GUS*-overexpressing hairy root line 1; LCn: *ZmLC*-overexpressing hairy root lines. Asterisks represent statistical significance (* *p* < 0.05; ** *p* < 0.01; *** *p* < 0.001).

**Figure 4:**
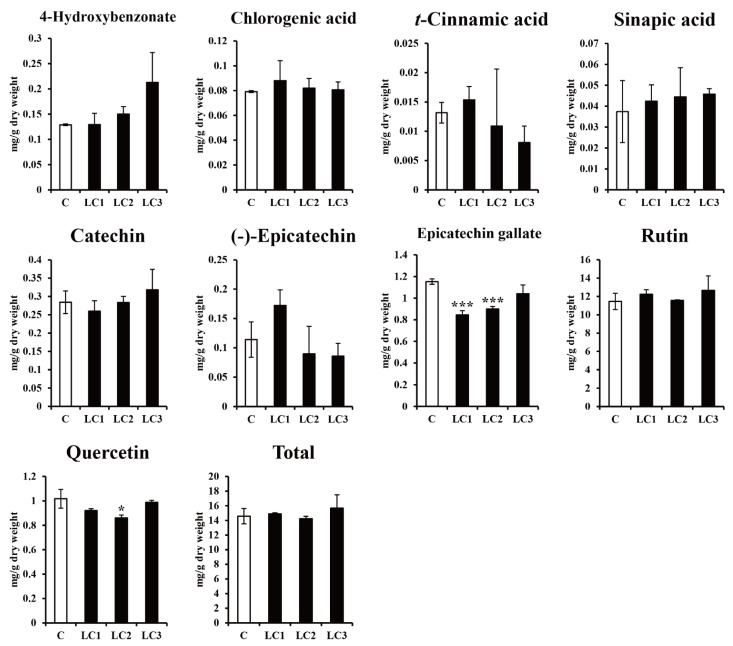
Effect of dark treatment on the phenolic compound accumulation of hairy root cultures in *Fagopyrum tataricum*. C: *GUS*-overexpressing hairy root line 1; LCn: *ZmLC*-overexpressing hairy root lines. Asterisks represent statistical significance (* *p* < 0.05; *** *p* < 0.001).

Three *ZmLC*- and one *GUS*-transgenic hairy root line (control) of Tartary buckwheat with fresh weights that were not significantly different were cultured in continuous darkness at 25 °C for two weeks (Table 1). The expression of 12 phenylpropanoid and flavonoid biosynthetic genes (*FtPAL*, *FtC4H*, *Ft4CL*, *FtCHS*, *FtCHI*, *FtF3H*, *FtF3′H1*, *FtFLS2*, *FtDFR*, *FtLAR*, *FtANS*, and *FtANR*) was assessed in the four lines, revealing that *FtCHI*, *FtF3H*, and *FtANS* expression was slightly higher in controls, but the expression of most genes was not significantly different (Figure 3). As shown in Figure 4, nine phenolic compounds (4-hydroxybenzonate, chlorogenic acid, *t*-cinnamic acid, sinapic acid, catechin, (−)-epicatechin, epicatechin gallate, rutin, and quercetin) were detected in three *ZmLC*- and one *GUS*-transgenic hairy root lines of Tartary buckwheat. The level of epicatechin gallate was slightly higher in the *GUS*-transgenic hairy root line than in the three *ZmLC*-transgenic lines; in addition, the total content (sum of the nine phenolics) was not significantly different among the transgenic hairy root lines. The phenolic contents of the transgenic hairy roots cultured in continuous darkness were consistent with the results of gene expression analysis.

### 2.3. Effect of Light Treatment on the Production of Phenolic Compound in ZmLC- and GUS-Transgenic Hairy Root Lines of Tartary Buckwheat

**Figure 5:**
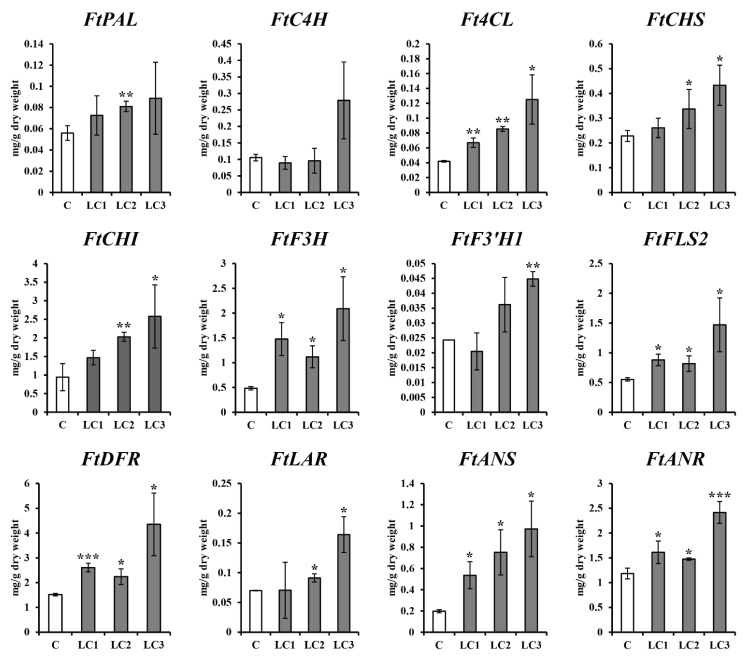
Effect of light treatment on the expression profiles of hairy root cultures in *Fagopyrum tataricum*. C: GUS-overexpressing hairy root line 1; LCn: ZmLc-overexpressing hairy root lines. Asterisks represent statistical significance (* *p* < 0.05; ** *p* < 0.01; *** *p* < 0.001).

**Figure 6:**
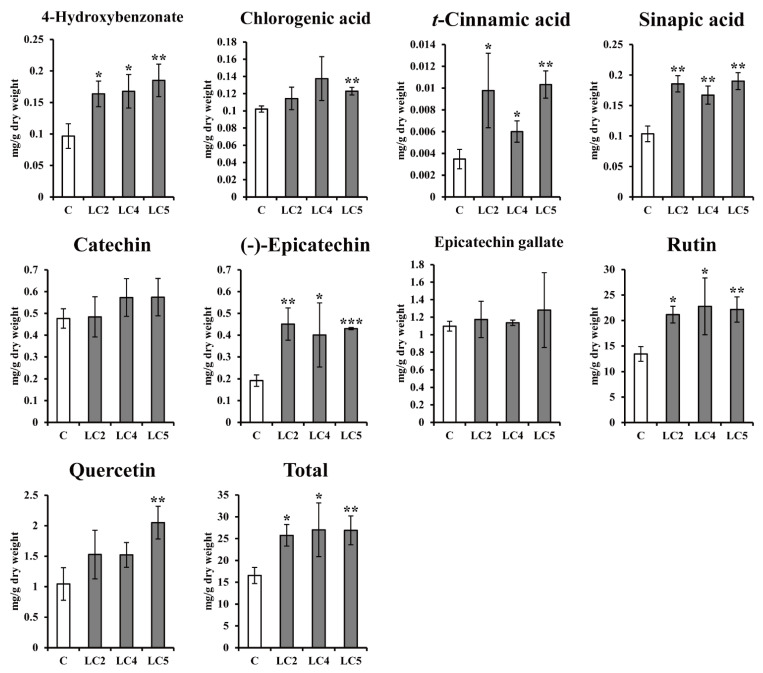
Effect of light treatment on the phenolic compound accumulation of hairy root cultures in *Fagopyrum tataricum*. C: *GUS*-overexpressing hairy root line 1; LCn: *ZmLC*-overexpressing hairy root lines. Asterisks represent statistical significance (* *p* < 0.05; ** *p* < 0.01; *** *p* < 0.001).

Three *ZmLC*- and one *GUS*-transgenic hairy root line (control) of Tartary buckwheat were cultured at 25 °C under a long photoperiod (16 h light and 8 h dark) for two weeks. The fresh weights of the transgenic lines were not significantly different (Table 2). According to the gene expression profiles, the majority of genes were likely to be more highly expressed in the *ZmLC*-transgenic hairy roots than in controls. The expression of *Ft4CL*, *FtF3H*, *FtFLS2*, *FtDFR*, *FtANS*, and *FtANR* was higher in the *ZmLC*-transgenic hairy roots in particular (Figure 5). The HPLC analysis showed an increase in phenolic compounds in *ZmLC*-transgenic hairy root lines in comparison with controls, which was consistent with the profiles in Figure 6. In particular, the total content (sum of nine phenolics) was 1.32-, 1.60-, and 1.61-fold higher in the three *ZmLC*-transgenic hairy root lines than the controls, and greater concentrations of 4-hydroxybenzonate, *t*-cinnamic acid, sinapic acid, and rutin were also shown in these lines.

## 3. Discussion

In this study, the transgenic hairy root lines of Tartary buckwheat were analyzed for phenolic compounds, including 4-hydroxybenzonate, chlorogenic acid, *t*-cinnamic acid, sinapic acid, catechin, (−)-epicatechin, epicatechin gallate, rutin, and quercetin. Previous data have revealed that the hairy roots of Tartary buckwheat can be a rich source of many phenolics, such as rutin, quercetin, (−)-epicatechin, catechin, and chlorogenic acid, which were identified in the hairy and seedling roots of Tartary buckwheat by Kim et al. [22]. Similarly, Thew et al. described rutin, quercetin, and 4-hydroxybenzonate in Hokkai T10 hairy roots [23], and Kim et al. detected 4-hydroxybenzonate, chlorogenic acid, *t*-cinnamic acid, rutin, and quercetin in Tartary buckwheat plantlets [24]. The results obtained in this study indicated that continuous darkness treatment did not affect the phenylpropanoid and flavonoid biosynthesis in the *GUS*- and three *ZmLC*-transgenic hairy root lines of Tartary buckwheat, whereas the long-photoperiod treatment enhanced the biosynthesis of these lines. Therefore, it is suggested that the effect of *ZmLC* on the accumulation of phenolics, including flavonoids, was light-induced, which is corroborated by previous studies describing the exogenous expression of *ZmLC* in petunia [17,25], tomato [26], alfalfa [18], and cotton [19] exhibiting light-induced anthocyanin pigmentation. Specifically, Albert et al. [17] and Bradley et al. [25] reported that anthocyanin accumulation was improved in *ZmLC*-transgenic petunia plants grown under high light conditions. Tomato seedlings heterologously expressing *ZmLC* and *ZmC1* had three- to four-fold greater anthocyanin concentrations than wild-type seedlings under light stress [26]. Furthermore, *ZmLC* was highly expressed in *ZmLC*-overexpressing alfalfa leaves, whereas anthocyanin production was found only under high light intensity [18]. Fan et al. reported that *ZmLC*-transgenic cotton plants exhibited white fibers that changed to red after light treatment [19].

Many studies have focused on the enhancement of flavonoid production in bacteria [27,28], yeast [29], and plants [21]. Hairy roots from various plant species have been developed for the production of phenolic compounds because of their ability to produce large amounts of metabolites and their high growth rates [30]. Light intensity, irradiance (continuous irradiance or continuous darkness), and quality can be paramount in the greening, growth, and metabolite biosynthesis of hairy roots. Jacob and Malpathak reported that light irradiation turned the roots of *Acmella oppositifolia*, *Lippia dulcis*, and *Datura stramonium* green and increased their phytochemical yield [31]. Light significantly increased the concentration of rutin in the hairy roots of *F. tataricum* Hokkai T10, and baicalein in hairy root cultures of *Scutellaria lateriflora* compared to the dark-grown roots of these species [23,32]. Abbasi et al. exposed the hairy roots of *Echinacea purpurea* to 50-days of continuous light (60 μmol·s^−1^·m^−2^) and found increased concentrations of anthocyanins caffeic acid, chlorogenic acid, and cichoric acid compared with the samples in continuous darkness [33]. Liu et al. reported a greater artemisinin yield in *Artemisia annua* hairy roots with an increase in light intensity of up to 3000 lux [34]. In this study, phenylpropanoid and flavonoid biosynthesis were upregulated under a relatively low light intensity (30 μmol·s^−1^·m^−2^) in Hokkai T10 hairy roots overexpressing *ZmLC*. Therefore, further research on the optimization of light intensity to improve the phenolic compound production in transgenic hairy roots should be performed.

## 4. Materials and Methods

### 4.1. Chemicals 

HPLC grade methanol were purchased from Samchun Pure Chemical, Pyeongtaek, Korea. Ethanol, NaClO, and acetic acid were purchased from Daejung, Siheung, Korea. LB broth, SH medium, cefotaxime, and kanamycin were purchased from Kisanbio, Seoul, Korea.

### 4.2. Plant Materials

Hokkai T10 seeds were obtained from the National Agricultural Research Center (Hokkaido, Japan). The seeds were soaked in 70% aqueous ethanol (*v/v*) for 1 min, followed by 4% NaClO for 10 min. After rinsing the seeds with sterilized water five times, they were incubated on half-strength SH solid medium (pH 5.8) in a growth chamber at 25 °C under normal white fluorescent bulbs with a flux rate of 30 μmol·s^−1^·m^−2^ and a long photoperiod (16 h light and 8 h dark) for 3 weeks, in preparation for hairy root induction.

### 4.3. Hairy Root Induction

The protocol for hairy root induction was that of Thew et al. [20]. *Agrobacterium rhizogenes* R1000 strains harboring pB7FWG2, *ZmLC*, or GUS pB7FWG2, were incubated in 30 mL of LB broth at 180 rpm and 28 °C for 1 d. After centrifugation (A_600_ = 0.6) and removal of the supernatant, the cell pellet was resuspended in SH liquid medium. The buckwheat seedlings were cut to the appropriate size in a Petri dish (Hyundai Micro, Seoul, Korea), containing the *A. rhizogenes* suspension and incubated for 20 min, followed by drying by patting with sterile paper for suspension removal. Subsequently, the explants were loaded onto an SH solid medium and then co-cultured in continuous darkness at 25 °C. After 2 days, the explants were rinsed with autoclaved water and then patted dry with sterile paper. They were then incubated on a half-strength SH solid medium containing 500 mg/L cefotaxime and 50 g/mL kanamycin. Hairy roots began to appear at the infected sites of the explants within 3 weeks, at which time the individual hairy roots were cultured on a half-strength SH solid medium containing 500 mg/L cefotaxime and 50 g/mL kanamycin at 25 °C in continuous darkness for 4 weeks. After transferring each hairy root onto fresh SH solid medium, the roots were cultured for an additional 4 weeks. Five grams of each hairy root line was then suspended in 30 mL of half-strength SH liquid medium at 110 rpm and 25 °C in continuous darkness for 2 weeks. The *ZmLC*- or *GUS*-transgenic hairy root lines were harvested and ground with liquid nitrogen. Some of the sample powders were used for DNA and RNA extraction, and the remaining samples were freeze-dried for secondary metabolite analysis.

### 4.4. Extraction of Genomic DNA and Polymerase Chain Reaction (PCR) Analysis

Genomic DNA of three *ZmLC*-transgenic lines and one *GUS*-transgenic hairy root line of Tartary buckwheat were isolated using a plant DNA extraction kit (Geneaid Biotech Ltd., Taipei, Taiwan). The primers for *rol* A–D and *bar* genes and conditions for the PCR reaction were based on a previous study [35]. PCR products of the expected lengths (500, 360, 900, 514, and 1035 bp) of *bar* and *rol* A, B, C, and D, respectively, were separated on a 1% agarose gel (data not shown).

### 4.5. Extraction of Total RNA and cDNA Synthesis

Total RNA of three *ZmLC*-transgenic lines and one GUS-transgenic hairy root line of Tartary buckwheat was isolated using the CTAB method combined with the RNeasy Plant Mini Kit (QIAGEN, Valencia, CA, USA). The quantity of RNA extracted from each line was measured using a NanoVue Plus spectrophotometer (GE Healthcare, Buckinghamshire, UK), and the quality of RNA was assessed by agarose gel electrophoresis. One microgram of each hairy root line RNA was reverse-transcribed using a First Strand Synthesis Kit (Toyobo, Osaka, Japan) and PCR according to the manufacturer’s instructions. After a twenty-fold dilution of each cDNA, quantitative real-time PCR (qRT-PCR) was performed.

### 4.6. Gene Expression Analysis

A CFX96 Real-Time System combined with a C1000 Thermal Cycler (Bio-Rad, Hercules, CA, USA) was used with gene-specific primers to express *FtPAL*, *FtC4H*, *Ft4CL*, *FtCHS*, *FtCHI*, *FtF3H*, *FtF3′H1*, *FtFLS2*, *FtDFR*, *FtLAR*, and *FtANS* in three *ZmLC*-transgenic and one *GUS*-transgenic hairy root line of Tartary buckwheat, as previously reported by Li et al. [21]. The reaction was started with a pre-denaturation step at 95 °C for 15 min, followed by 40 cycles of denaturation at 95 °C for 15 s, primer annealing at 60 °C for 15 s, extension at 72 °C for 20 s, and final amplification at 72 °C for 15 min. Three technical and biological replicates of the four lines of Tartary buckwheat were used for the reaction.

### 4.7. HPLC Analysis of Phenolic Compounds

HPLC analysis of phenolic compounds in *ZmLC*- and *GUS*-transgenic hairy root lines of Tartary buckwheat was performed according to the method reported by Park et al. [35]. Briefly, 1.5 mL of 80% (*v/v*) aqueous methanol was added to a tube containing 100 mg of three *ZmLC*-transgenic and one *GUS*-transgenic hairy root line and vortexed for 1 min. After sonication for 1 h and centrifugation at 11,000 × *g* at 4 °C for 20 min, the supernatant was syringe-filtered into a vial. Phenolics were separated using methanol (A) and 0.2% acetic acid-water (B). The HPLC system (NS-4000, Futecs Co., Daejeon, Korea), column (OptimaPak, 250 × 4.6 mm, 5 µm, RStech Co., Daejeon, Korea), and analysis conditions were obtained from a previous study [35]. Comparison of retention time and spiking tests were performed to identify the phenolics, and the calibration curves for each phenolic compound were used to determine the concentration in the samples.

## 5. Conclusions

This is the first study on the effect of continuous light and darkness on the accumulation of phenolic compounds, including rutin, in *ZmLC*-transgenic hairy roots through the enhancement of phenylpropanoid and flavonoid biosynthesis in hairy roots cultured under continuous light. Furthermore, this study suggested that Tartary buckwheat Hokkai T10 can be a viable plant source for the application of metabolic engineering with the aim of overproducing beneficial metabolites.

## Figures and Tables

**Table 1:** Fresh weight (g) of *GUS*- and *ZmLC*-overexpressing hairy root lines cultured at 25 °C in continuous darkness for two weeks.

Line	Fresh Weight (g)
C ^1^	1.52 ± 0.10
LC1 ^2^	1.83 ± 0.45
LC2	1.69 ± 0.18
LC3	2.04 ± 0.21

^1^ C: *GUS*-overexpressing hairy root line; ^2^ LCn: *ZmLC*-overexpressing hairy root lines.

**Table 2:** Fresh weight (g) of *GUS*- and *ZmLC*-overexpressing hairy root lines cultured at 25 °C in the long-photoperiod condition for two weeks.

Line	Fresh Weight (g)
C ^1^	1.83 ± 0.86
LC1 ^2^	3.25 ± 1.25
LC2	2.45 ± 0.95
LC3	2.11 ± 0.57

^1^ C: *GUS*-overexpressing hairy root line; ^2^ LCn: *ZmLC*-overexpressing hairy root lines.

## References

[1] Bonafaccia G., Marocchini M., Kreft I. (2003). Composition and technological properties of the flour and bran from common and tartary buckwheat. Food Chem..

[2] Kawa J.M., Taylor C.G., Przybylski R. (2003). Buckwheat concentrate reduces serum glucose in streptozotocin-diabetic rats. J. Agric. Food Chem..

[3] Chan P.-K. (2003). Inhibition of tumor growth in vitro by the extract of fagopyrum cymosum (fago-c). Life Sci..

[4] Ujita M., Nagayama H., Kanie S., Koike S., Ikeyama Y., Ozaki T., Okumura H. (2009). Carbohydrate binding specificity of recombinant human macrophage β-glucan receptor dectin-1. Biosci. Biotechnol. Biochem..

[5] Hu Z.B., Du M. (2006). Hairy root and its application in plant genetic engineering. J. Integr. Plant Biol..

[6] Shanks J.V., Morgan J. (1999). Plant ‘hairy root’culture. Curr. Opin. Biotechnol..

[7] Kim Y.J., Wyslouzil B.E., Weathers P.J. (2002). Secondary metabolism of hairy root cultures in bioreactors. In Vitro Cell. Dev. Biol. Plant.

[8] Makhzoum A.B., Sharma P., Bernards M.A., Trémouillaux-Guiller J., David R.G. (2013). Hairy roots: An ideal platform for transgenic plant production and other promising applications. Phytochemicals, Plant Growth, and the Environment.

[9] Ludwig S.R., Habera L.F., Dellaporta S.L., Wessler S.R. (1989). Lc, a member of the maize R gene family responsible for tissue-specific anthocyanin production, encodes a protein similar to transcriptional activators and contains the myc-homology region. Proc. Natl. Acad. Sci. USA.

[10] Huang Z.-A., Zhao T., Wang N., Zheng S.-S. (2016). Ectopic expression of Lc differentially regulated anthocyanin biosynthesis in the floral parts of tobacco (*Nicotiana tobacum* L.) plants. Bot. Stud..

[11] Li H.H., Flachowsky H., Fischer T.C., Hanke M.-V., Forkmann G., Treutter D., Schwab W., Hoffmann T., Szankowski I. (2007). Maize Lc transcription factor enhances biosynthesis of anthocyanins, distinct proanthocyanidins and phenylpropanoids in apple (*Malus domestica* Borkh.). Planta.

[12] Park C.H., Xu H., Yeo H.J., Park Y.E., Hwang G.-S., Park N.I., Park S.U. (2021). Enhancement of the flavone contents of *Scutellaria baicalensis* hairy roots via metabolic engineering using maize Lc and Arabidopsis PAP1 transcription factors. Metab. Eng..

[13] Boase M.R., Bradley J.M., Borst N.K. (1998). Genetic transformation mediated by *Agrobacterium tumefaciens* of florists’ chrysanthemum (*Dendranthema xgrandiflorum*) cultivar ‘Peach Margaret’. In Vitro Cell. Dev. Biol. Plant.

[14] Bradley J.M., Deroles S.C., Boase M.R., Bloor S., Swinny E., Davies K.M. (1999). Variation in the ability of the maize Lc regulatory gene to upregulate flavonoid biosynthesis in heterologous systems. Plant Sci..

[15] Han Y.-J., Kim Y.-M., Lee J.-Y., Kim S.J., Cho K.-C., Chandrasekhar T., Song P.-S., Woo Y.-M., Kim J.-I. (2009). Production of purple-colored creeping bentgrass using maize transcription factor genes *Pl* and *Lc* through *Agrobacterium*-mediated transformation. Plant Cell Rep..

[16] Procissi A., Dolfini S., Ronchi A., Tonelli C. (1997). Light-dependent spatial and temporal expression of pigment regulatory genes in developing maize seeds. Plant Cell.

[17] Albert N.W., Lewis D.H., Zhang H., Irving L.J., Jameson P.E., Davies K. (2009). Light-induced vegetative anthocyanin pigmentation in Petunia. J. Exp. Bot..

[18] Ray H., Yu M., Auser P., Blahut-Beatty L., McKersie B., Bowley S., Westcott N., Coulman B., Lloyd A., Gruber M.Y. (2003). Expression of anthocyanins and proanthocyanidins after transformation of alfalfa with maize Lc. Plant Physiol..

[19] Fan X., Fan B., Wang Y., Yang W. (2016). Anthocyanin accumulation enhanced in *Lc*-transgenic cotton under light and increased resistance to bollworm. Plant Biotechnol. Rep..

[20] Thwe A., Valan Arasu M., Li X., Park C.H., Kim S.J., Al-Dhabi N.A., Park S.U. (2016). Effect of different *Agrobacterium rhizogenes* strains on hairy root induction and phenylpropanoid biosynthesis in tartary buckwheat (*Fagopyrum tataricum* Gaertn). Front. Microbiol..

[21] Li X., Sathasivam R., Park N.I., Wu Q., Park S.U. (2020). Enhancement of phenylpropanoid accumulation in tartary buckwheat hairy roots by overexpression of MYB transcription factors. Ind. Crops Prod..

[22] Kim Y.K., Li X., Xu H., Park N.I., Uddin M.R., Pyon J.Y., Park S.U. (2009). Production of phenolic compounds in hairy root culture of tartary buckwheat (*Fagopyrum tataricum* Gaertn). J. Crop Sci. Biotech..

[23] Thwe A.A., Kim Y.J., Li X., Kim Y.B., Park N.-I., Kim H.H., Kim S.-J., Park S.U. (2014). Accumulation of phenylpropanoids and correlated gene expression in hairy roots of tartary buckwheat under light and dark conditions. Appl. Biochem. Biotechnol..

[24] Kim N.S., Kwon S.-J., Cuong D.M., Jeon J., Park J.S., Park S.U. (2019). Accumulation of phenylpropanoids in tartary buckwheat (*Fagopyrum tataricum*) under salt stress. Agronomy.

[25] Bradley J.M., Davies K.M., Deroles S.C., Bloor S.J., Lewis D.H. (1998). The maize *Lc* regulatory gene up-regulates the flavonoid biosynthetic pathway of Petunia. Plant J..

[26] Bovy A., de Vos R., Kemper M., Schijlen E., Pertejo M.A., Muir S., Collins G., Robinson S., Verhoeyen M., Hughes S. (2002). High-flavonol tomatoes resulting from the heterologous expression of the maize transcription factor genes *LC* and *C1*. Plant Cell.

[27] Li J., Tian C., Xia Y., Mutanda I., Wang K., Wang Y. (2019). Production of plant-specific flavones baicalein and scutellarein in an engineered *E. coli* from available phenylalanine and tyrosine. Metab. Eng..

[28] Solopova A., van Tilburg A.Y., Foito A., Allwood J.W., Stewart D., Kulakauskas S., Kuipers O.P. (2019). Engineering *Lactococcus lactis* for the production of unusual anthocyanins using tea as substrate. Metab. Eng..

[29] Sáez-Sáez J., Wang G., Marella E.R., Sudarsan S., Pastor M.C., Borodina I. (2020). Engineering the oleaginous yeast *Yarrowia lipolytica* for high-level resveratrol production. Metab. Eng..

[30] Park C.H., Zhao S., Yeo H.J., Park Y.E., Baska T.B., Arasu M.V., Al-Dhabi N.A., Park S.U. (2017). Comparison of Different Strains of *Agrobacterium rhizogenes* for Hairy Root Induction and Betulin and Betulinic Acid Production in Morus alba. Nat. Prod. Commun..

[31] Jacob A., Malpathak N. (2004). Green hairy root cultures of *Solanum khasianum* Clarke—A new route to in vitro solasodine production. Curr. Sci..

[32] Marsh Z., Yang T., Nopo-Olazabal L., Wu S., Ingle T., Joshee N., Medina-Bolivar F. (2014). Effect of light, methyl jasmonate and cyclodextrin on production of phenolic compounds in hairy root cultures of *Scutellaria lateriflora*. Phytochem.

[33] Abbasi B.H., Tian C.-L., Murch S.J., Saxena P.K., Liu C.-Z. (2007). Light-enhanced caffeic acid derivatives biosynthesis in hairy root cultures of *Echinacea Purpurea*. Plant Cell Rep..

[34] Liu C.-Z., Guo C., Wang Y.-C., Ouyang F. (2002). Effect of light irradiation on hairy root growth and artemisinin biosynthesis of *Artemisia Annu*. Process Biochem..

[35] Cuong D.M., Park C.H., Bong S.J., Kim N.S., Kim J.K., Park S.U. (2019). Enhancement of glucosinolate production in watercress (*Nasturtium officinale*) hairy roots by overexpressing cabbage transcription factors. J. Agric. Food Chem..

